# Rationale and design of the participant, investigator, observer, and data-analyst-blinded randomized AGENDA trial on associations between gene-polymorphisms, endophenotypes for depression and antidepressive intervention: the effect of escitalopram versus placebo on the combined dexamethasone-corticotrophine releasing hormone test and other potential endophenotypes in healthy first-degree relatives of persons with depression

**DOI:** 10.1186/1745-6215-10-66

**Published:** 2009-08-11

**Authors:** Ulla Knorr, Maj Vinberg, Marianne Klose, Ulla Feldt-Rasmussen, Linda Hilsted, Anders Gade, Eva Haastrup, Olaf Paulson, Jørn Wetterslev, Christian Gluud, Ulrik Gether, Lars Kessing

**Affiliations:** 1 Department of Psychiatry, Rigshospitalet, Copenhagen University Hospital, Copenhagen, Denmark; 2 Department of Medical Endocrinology, Rigshospitalet, Copenhagen University Hospital, Copenhagen, Denmark; 3 Department of Biochemistry, Rigshospitalet, Copenhagen University Hospital, Copenhagen, Denmark; 4 Institute of Psychology, University of Copenhagen, Copenhagen, Denmark; 5 Department of Clinical Immunology, Centre of Clinical Investigation, Rigshospitalet, Copenhagen University Hospital, Copenhagen, Denmark; 6 Danish Research Centre for Magnetic Resonance, Hvidovre Hospital, Copenhagen University Hospital, Copenhagen, Denmark; 7 Neurobiology Research Unit, Copenhagen University Hospital Rigshospitalet, Denmark; 8 Centre for Integrated Molecular Brain Imaging, Copenhagen, Denmark; 9 Copenhagen Trial Unit, Centre for Clinical Intervention Research, Rigshospitalet, Copenhagen University Hospital, Denmark; 10 Department of Neuroscience and Pharmacology, University of Copenhagen, Copenhagen, Denmark

## Abstract

**Background:**

Endophenotypes are heritable markers, which are more prevalent in patients and their healthy relatives than in the general population. Recent studies point at disturbed regulation of the hypothalamic-pituitary-adrenocortical axis as a possible endophenotype for depression. We hypothesize that potential endophenotypes for depression may be affected by selective serotonin re-uptake inhibitor antidepressants in healthy first-degree relatives of depressed patients. The primary outcome measure is the change in plasma cortisol in the dexamethasone-corticotrophin releasing hormone test from baseline to the end of intervention.

**Methods:**

The AGENDA trial is designed as a participant, investigator, observer, and data-analyst-blinded randomized trial. Participants are 80 healthy first-degree relatives of patients with depression. Participants are randomized to escitalopram 10 mg per day versus placebo for four weeks. Randomization is stratified by gender and age. The primary outcome measure is the change in plasma cortisol in the dexamethasone-corticotrophin releasing hormone test at entry before intervention to after four weeks of intervention. With the inclusion of 80 participants, a 60% power is obtained to detect a clinically relevant difference in the primary outcome between the intervention and the placebo group. Secondary outcome measures are changes from baseline to four weeks in scores of: 1) cognition and 2) neuroticism. Tertiary outcomes measures are changes from baseline to four weeks in scores of: 1) depression and anxiety symptoms; 2) subjective evaluations of depressive symptoms, perceived stress, quality of life, aggression, sleep, and pain; and 3) salivary cortisol at eight different timepoints during an ordinary day. Assessments are undertaken by assessors blinded to the randomization group.

**Trial registration:**

Local Ethics Committee: H-KF 307413

Danish Medicines Agency: 2612-3162.

EudraCT: 2006-001750-28.

Danish Data Agency: 2006-41-6737.

ClinicalTrials.gov: NCT 00386841

## Background

Robins and Guze described five phases in the development of a valid classification of psychiatric illness: clinical description, laboratory studies, delimitation from other disorders, follow-up studies and family studies [1]. Later, response to treatment was added as a sixth phase [2]. Recently, the endophenotype concept has emerged as a strategic tool in neuropsychiatric research [3].

Endophenotypes are quantifiable components in the "genes-to-behaviours" pathways distinct from psychiatric symptoms [3]. In parallel with the classification of psychiatric diseases, endophenotypes are validated by specificity, state independence, heritability, familial association, co-segregation, and biological and clinically plausibility [4].

Several possible endophenotypes have been proposed in affective disorders, including stress regulation, cognition, neuroticism, depression and anxiety symptoms [4]. Pharmacological anti-depressants may have an effect on endophenotypes in healthy persons with a family history of depression. We hypothesized that treatment response could be added to the validation of possible endophenotypes for depression. However, a systematic search for randomized multiple-dose, placebo-controlled trials on the effect of selective serotonin reuptake inhibitors did not identify any trials in which healthy first-degree relatives of depressed patients received a selective serotonin re-uptake inhibitor for at least one week (unpublished data).

### Possible endophenotypes for depression

#### Hypothalamus-pituitary-adreno-cortical (HPA) axis regulation

Impaired regulation of the HPA axis during an acute episode of depression is the most consistent laboratory finding [5-7]. The combined dexamethasone (DEX)-corticotrophine releasing hormone (CRH) test is a sensitive test for detecting altered HPA axis regulation [8]. In this test, the stimulating effects of 100 μg CRH on corticotrophin (ACTH) and cortisol are examined under the suppressive action of 1.5 mg of dexamethasone. ACTH and cortisol in the combined DEX-CRH test demonstrated an exaggerated response in patients with depression compared to healthy controls with a family history of depression [9] and between healthy first-degree relatives of patients with depression compared to healthy controls without a family history of depression [9]. Increased ACTH and cortisol in the combined DEX-CRH test therefore seems to be a promising biomarker for depression and a potential endophenotype for depression [10].

#### Cognition

Alterations in cognitive functions are common and included in the diagnostic criteria for depression [11] and some patients experience cognitive dysfunction even in euthymic phases of the disease [12-16]. A high-risk study showed impairment of selective and sustained attention, executive function, language processing and working and declarative memory in subjects with a family history of depression as compared to participants without [17]. Thus cognitive function may be a candidate endophenotype for affective disorders.

#### Personality

Neuroticism is a measure of an individual's tendency to experience negative emotions that are manifested at one extreme as anxiety, depression, and moodiness, and at another extreme, as emotional stability. Neuroticism is most frequently measured by questionnaires such as Eysenck Personality Questionnaire (EPQ) [18] and the revised NEO Personality Inventory (NEO-PI-R) [19]. The heritability of neuroticism is well established [20]. Studies of healthy first-degree relatives of patients with a depression point at neuroticism as an endophenotype for depression [21].

#### Other potential endophenotypes for depression

More than 90% of depressed patients complain about impairment of sleep quality, which has been suggested as a potential endophenotype for depression [22]. The effect of antidepressants on sleep quality in healthy individuals with a family history of depression has not been investigated. Subjective measures of stress, aggression, pain, and quality of life are all factors known to improve with remission of depressive symptoms. Whether this is a direct effect of treatment with antidepressants or a consequence of improvement in depressive symptoms is unclear.

### Proportion of possible endophenotypes for depression

Based on results from recent studies it is estimated that 30% of healthy persons with a family history of depression will exhibit at least two of the three possible endophenotypes: dysregulation of the HPA axis, cognition, and neuroticism [23]. The prevalence of other possible endophenotypes is unknown.

### The effect of antidepressants on endophenotypes for depression

Treatment with antidepressants in patients with an acute depression is associated with partial normalization of the HPA axis [24,25], enhanced cognitive function [26], and reduction in the personality trait of neuroticism [27]. In these trials it has not been possible to distinguish the treatment effect on the endophenotypes from the treatment effect on the disease, since remission of depressive symptoms is associated with partial normalization of the endophenotypes. It is not known whether the treatment responses on the disease symptoms are mediated through an effect on the endophenotypes or vice versa. Results of recent randomized, placebo-controlled trials suggest that antidepressants have an effect on psychological variables and behaviour in individuals without psychiatric illness [28-30], one of these studies did not mention the family history status of the included individuals [28] but in two of the studies [29,30] healthy individuals with a family history of psychiatric illness were excluded.

In summary, no trial has investigated the effects of antidepressants on possible endophenotypes in healthy individuals with a family history of depression.

### Genotyping

A recent systematic review and meta-analysis of pharmacogenetic studies of antidepressant response suggests that polymorphisms in genes such as 5-HTTLPR, STin2, HTR1A, HTR2A, TPH1, and BDNF may modulate antidepressant response [31], but the association between gene polymorphisms and the effect of an antidepressant treatment on the putative endophenotypes for depression has not been explored.

### Objectives

With the AGENDA trial (**A**ssociations between **G**ene-polymorphisms, **E**ndophenotypes for **D**epression and **A**ntidepressive Intervention) we want to test the hypothesis that potential endophenotypes for depression are affected by intervention with an antidepressant in healthy first-degree relatives of patients with the diagnosis of depression.

## Methods

**Figure 1:**
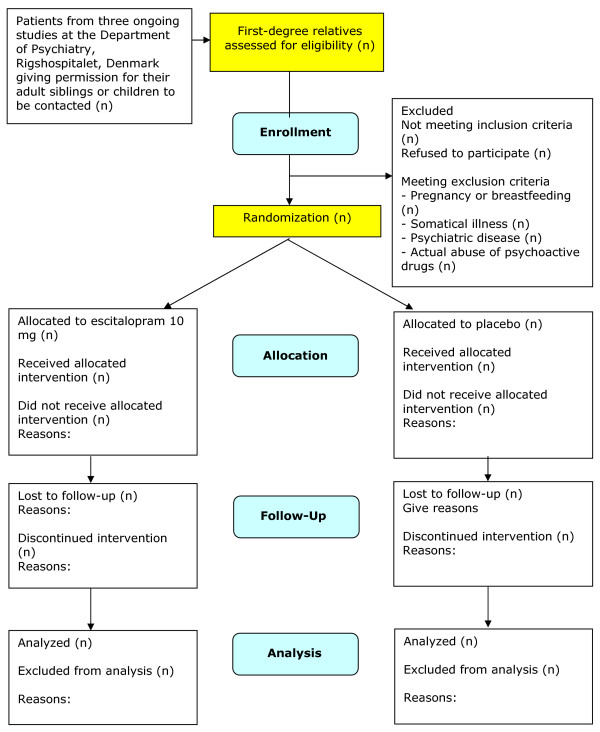
**Flowchart for The Agenda Trial**.

The AGENDA trial is designed as a participant, investigator, observer, and data-analyst-blinded randomized trial in which participants receive either escitalopram 10 mg or placebo for a period of four weeks (Figure 1).

### Study organization

The study is conducted at the Department of Psychiatry, Rigshospitalet, Copenhagen University Hospital, Denmark as part of the Centre for Pharmacogenomics, University of Copenhagen. The trial has a data monitoring and safety committee (DMSC) that is independent of the investigators conducting the trial.

### Participants

Participants are recruited as healthy first-degree relatives of patients with a diagnosis of depression given at discharge from psychiatric hospital in- or out-patient contact [32]. These patients participated in ongoing studies at the psychiatric department of Rigshospitalet, University of Copenhagen, Denmark. Individuals meeting the inclusion criteria and none of the exclusion criteria are enrolled in the trial (Appendix 1). The exclusion criteria were chosen partly for safety reasons and partly to decrease the risk of results being confounded by factors known to substantially affect the HPA-axis, thus interfering the primary outcome measure. Women taking birth control pills were instructed to discontinue these 6 weeks prior to entering the trial. All women were carefully instructed to use double barrier birth control methods and pregnancy tests were performed both before and after the intervention.

### Interventions

The participants are randomized to receive either escitalopram or placebo by oral administration as a single dose of 10 mg each evening as self-medication at home for four weeks. On completion of four weeks of double-blind intervention (or early discontinuation from the trial) participants entered a five-day blinded down-titration period to nil medication. Escitalopram 10 mg was selected because of its specific serotonergic selectivity [33] and because of the favourable adverse reaction profile [34], thus facilitating blinding. The dose of escitalopram 10 mg for four weeks was estimated to have a sufficient effect, since effect on depressive symptoms in patients with a major depression is observed within one to four weeks as compared with placebo [34]. The validity of the results depends on a high compliance and high completion in the trial. This was sought obtained by weekly telephone control calls to the enrolled participants to insure adherence to the protocol and to record adverse events. Escitalopram and placebo tablets were identical in appearance, colour, smell, and solubility allowing for blinding of treatment assignment. H. Lundbeck A/S provided identically appearing blister packages containing escitalopram or placebo. An independent pharmacist then packed, sealed and numbered the drug packages according to a randomization list provided and concealed by the Copenhagen Trial Unit (CTU), Centre for Clinical Intervention Research, Rigshospitalet.

### Randomization

Randomization into one of the two intervention groups is done immediately after it has been established that the participant fulfils all the inclusion criteria and none of the exclusion criteria. Randomization is stratified by age (18 – 31 years and 32 – 60 years) and sex in order to get an equal distribution in the intervention groups, knowing that the response to the DEX-CRH test is sensitive to these factors. Participants are randomized in a 1-to-1 ratio to receive escitalopram 10 mg or placebo. The Copenhagen Trial Unit (CTU), Centre for Clinical Intervention Research, Rigshospitalet performs the centralised randomization, and only the IT Manager of the CTU will know the block size used for stratification. The sponsor-investigator (UK) provides information of the participants to the CTU during the entry assessment as soon as participation in the study has been decided. CTU performs the computer-generated randomization to ensure adequate allocation concealment and adequate generation of the allocation sequence [35]. The number of the allocated treatment is communicated to UK, both by phone and email.

### Blinding

All study personnel and participants are blinded to the packaging of the study drug, and blinding is maintained throughout monitoring, follow-up, data management, assessment of outcomes and data analyses. The randomization code will not be broken until all the data has been analysed and conclusions drawn. At the assessment after four weeks intervention, every participant, the sponsor-investigator (UK), and the neuropsychological testers will make a guess as to which intervention the participant has received. When the trial is finished, inter-rater reliability between the actual intervention and the guesses will be estimated to assess the degree to which blinding has been successful.

### Outcome assessments

Outcomes are assessed at entry and after four weeks intervention by assessors who are all blinded to the randomization group.

Depression has a wide range of possible features that can be measured and tested as possible endophenotypes [36]. Since no prior trial has investigated the effect of SSRI on healthy first-degree relatives of patients with depression, we have chosen to include many outcomes since the nature of this trial is predominantly exploratory, i.e., hypothesis generating, and only partly hypothesis testing. When available, previously developed and validated scales and instruments were used in order to facilitate appropriate comparisons with data obtained in previous studies.

### Primary outcome

During selection of the primary outcome, it was stressed that the outcome should be objective and of clinical importance. Further, blinding should be possible at all levels of assessment and analyses. The measurement of change in plasma cortisol in the DEX-CRH test from entry before to four weeks during intervention fulfils these criteria. Plasma cortisol will be estimated as the total area under the curve (AUC-total) from administration of CRH at 15.00 hours to the last plasma cortisol measure at 18.00 hours. The DEX-CRH test is performed according to international standards with a few minor modifications [9]; thus plasma cortisol, plasma ACTH and salivary cortisol are measured at the same time points.

### Secondary outcomes

Secondary outcomes include changes in scores from baseline to four weeks on: 1) cognitive functions are measured with a broad battery of neuropsychological tests with relevance in relation to depression, evaluating memory, attention, visuomotor speed, visuo-constructional abilities, decision making, logical thinking, executive functions, verbal fluency, social and moral cognition, recognition of emotions and emotional intelligence [37-54], 2) neuroticism as measured by the NEO-PI-R [19] and EPQ [18].

### Tertiary outcomes

Tertiary outcomes include changes in scores from baseline to four weeks on: 1) mood as measured by the Hamilton rating scale for depression (HAM-D17) [55] and the Hamilton rating scale for anxiety (HAM-A14 [56], 2) participants subjective perception of pain on a visual analogue scale modified from Klepstad [57], 3) sleep on a visual analogue scale for sleep quality, sleep items from the HAMD-17 and supplementary questions on sleep characteristics, 4) aggression by The Buss-Perry Aggression Questionnaire [58], 5) depressive symptoms by the Beck Depression Inventory (BDI-21) [59], 6) quality of life by the WHO Quality of Life questionnaire [60], 7) a global measure of stress by the Cohen Perceived Stress Scale [61], and 8) salivary cortisol assessed by Salivettes during an ordinary day in the participants usual environment at the following time points: awakening, awakening + 15 min., awakening + 30 min., awakening + 45 min., awakening + 60 min., 12:00, 18:00 and at 23:00 [62]. Further, side-effects are assessed at four weeks by the UKU Side Effect Rating Scale [63].

### Genotyping

Blood samples are stored in a bio bank for further analyses. Among others we intend to genotype for the 5-HTTLPR-short/long-promoter variant, and the catechol-O-methyltransferase (COMT), and to test whether these genotypes will be associated with the response to escitalopram on the potential endophenotypes for depression. Further, messenger RNA for the glucocorticoid receptor will be analysed and associated to the effect of escitalopram.

### Assessments

Participants are subjected to almost identical sequences of assessments at entry and after four weeks of intervention (Appendix 2). The first part of the assessment is a telephone interview, and the individuals who are not excluded at that time point, are scheduled to meet at the clinic at two different days before and following four weeks of intervention with escitalopram or placebo. At the first day of examination the participants are interviewed to evaluate fulfilment of inclusion and exclusion criteria including the Schedules for Clinical Assessment in Neuropsychiatry (SCAN) [64] and various socio-demographics.

### Sample size

A high-risk study performed by Modell et al. [9] found that healthy high-risk probands of patients with a diagnosis of depression examined by the DEX-CRH test present with a cortisol AUC-total (mean ± SEM) of 15,064 ± 3,947 nmol × min/L. Further, Modell et al reported that cortisol AUC-total (mean ± SEM) in healthy individuals with no family history of depression was 7,773 ± 1,071 nmol × min/L. A clinically relevant effect of escitalopram on the cortisol AUC-total (mean ± SEM) was thus estimated to be the difference in cortisol AUC-total (mean ± SEM) of high-risk probands of patients with the diagnosis of depression and that of healthy individuals with no family history of depression. Accordingly, the relevant difference we aim to detect or reject is 15,064 – 7,773 = 7,291 nmol × min/L. Given a standard deviation (SD) = SEM × √14 = 3,947 × 3.7 = 14,768 nmol × min/L provides a power of the trial at a minimum of 60% (1 - β = 0.60), β being the risk of overlooking a difference in the cortisol AUC-total. However, the power in the trial may be higher, considering the use of analysis of covariance (ANCOVA) of the change from entry to after four weeks of intervention in AUC total during the DEX-CRH test. Based on these calculations we aim for a full data set of 80 participants to be able to conclude in relation to the primary outcome measure.

### Statistical methods

All data analyses will be carried out according to a pre-established analysis plan. The main null-hypothesis to be tested is that there is no difference between the two intervention arms with regard to the plasma cortisol AUC-total in the DEX-CRH test. All randomized participants will be analysed, including those with missing data on AUC.

Statistical analyses will be performed using ANCOVA [65]. Thus, the outcomes will be analysed as the difference for the individual participant's before and after the intervention, firstly unadjusted and then adjusted for a number of variables, if they present with a p-value < 0.1 in the univariate analyses [66], see Table 1. Initially the drug level measured in each participant will not be included in the models in order as to keep the analysers blinded. Later on analyses for the effect of drug-level will be performed along with other significant covariates in the multivariate model. Separate analyses will be performed in a log linear model of the changes in the primary outcome as compared to the secondary and tertiary outcomes.

**Table 1:** Covariates in the statistical models for the AGENDA trial.

Outcome	Primary outcome: AUC-total plasma cortisol	Secondary outcome Neuroticism	Secondary outcome Cognitive function	Tertiary outcomes
AUC-total plasma cortisol	x	x		x

Age	x	x	x	x

Gender	x	x	x	x

HAMD, entry	x		x	x

Body Mass Index, entry	x			

Number of daily cigarettes	x			

Danish Adult Reading Test			x	

Years of education		x		

Drug-level	x	x	x	x

In the case of missing data according to the primary outcome, analyses will be performed both on complete data sets, as well as data sets on all participants completed by multiple imputation (MI) of missing data by MI-analysis (SASS version 9.1 or NORM version 1) based on age, sex, body mass index, HAMD, neuroticism before and after the intervention, years of education, and AUC-total for cortisol, and ACTH and salivary cortisol before and after the intervention. In the case of discrepancy between these results, the result from the MI procedure is regarded as the result in the trial. P < 0.05 will be regarded as statistically significant.

### Data management

All the data of each participant is kept in a Case Record File, which fulfils the Danish law for medical doctors' obligation to keep patient records. In order to maintain blinding, the result of serum escitalopram concentration at end of the intervention is sent to the CTU, that keeps it in a locked safety box until the practical part and the data analyses of the trial are finished. Participants are not registered in The Danish Psychiatric Central Research Register or in any local hospital registers.

### Safety

Procedures for breaking the code for randomization has been established for the case of severe adverse reactions, which can be related to the intervention or if a serious adverse events occur. It is the decision of UK and LK to request emergency breaks, and the CTU can be contacted at any time regarding the practical procedure. The participants can at all times reach UK by mobile phone. An independent data monitoring and safety committee has been established to further ensure the safety of the participants, should the need for considering early stopping occur.

## Results

### Current trial status

Enrolment started July 2007 and is ongoing until July 2009. Status in May 2009 is that 390 eligible persons have been screened and that 77 of these have been randomized (Figure 1) and the dataset is complete for 64 participants regarding the primary outcome measure.

### Ethical considerations

The regional ethics committee for the greater Copenhagen area has approved the protocol (H-KF-307413) as has The Danish Data Protection Agency (2006-41-6737) and The Danish Medicines Agency (2612-3162). The trial has the EudraCT number 2006-001750-28 and is registered at ClinicalTrials.gov as NCT 00386841. Both positive, neutral, and negative findings from the trial will be published in accordance with the CONSORT guidelines [67]. The trial is conducted and monitored in accordance with the International Conference on Harmonization for Good Clinical Practice guidelines [66] and the Declaration of Helsinki 2002 .

Information about the trial is presented to potential participants both verbally and in written form in quiet surroundings, and the participants were given permission to bring a relative or friend. It is made clear that participation is voluntary and that the participant can withdraw the given consent at any time without consequence for future treatment possibilities. Participants receive a copy of their rights. All participating healthy volunteers sign a written informed consent. The participants are paid up to 9,000 Danish crowns for full participation (equal to about one weeks pay) and are further compensated for any travel expenses. When the randomization code is broken the participants will receive a letter with information on whether they received escitalopram or placebo and the major results of the trial.

## Discussion

The AGENDA trial is the first trial investigating whether an antidepressant has an effect on potential endophenotypes in healthy first-degree relatives of patients with a diagnosis of depression. This represents a new strategy to validate potential endophenotypes for depression, which may cast light on the pathophysiology of depression. The trial is fully investigator initiated and controlled to secure unbiased assessment of the effect of escitalopram on endophenotypes of healthy first-degree relatives of patients with depression. The AGENDA trial has received non-restricted grants from non-profit and for-profit organizations.

### Perspectives

Further knowledge of endophenotypes may increase the validity of the diagnosis of depression in the future and may eventually improve our possibilities to reclassify depression. The principle of testing the effect of a psychotropic drug on possible endophenotypes for a given disorder could be used to test putative endophenotypes for other disorders as well.

### Further outcome assessments

We plan to assess the effect of escitalopram versus placebo on a number of other outcomes in healthy participants with a family history of depression. These assessments include:

A: Investigation of the association between inflammation and depression. A flow-cytometric profile with focus on activated and non-activated t-cell subsets measured before and after intervention. Furthermore inflammatory variables and proinflammatory genepolymorphisms are measured.

B. Magnetic Resonance Imaging (MRI) of hippocampus volume. Studies of patients with unipolar depression suggest a decreased volume of hippocampus in MR scans [68]. Decreased volume of hippocampus is a possible endophenotype for depression and the effect of SSRI on hippocampal volume has not been established in healthy individuals with a family history of depression. Functional MRI including Face Emotion – Gender Discrimination Task [69] and Flanker Go-No go Risk paradigm [70] is conducted in a subpopulation of participants in the AGENDA trial.

## Competing interests

UK, MK, UFR, LH, AG, EH, OP, JW, and CG declare to have no competing interests.

MV has been a speaker for Eli Lilly, Wyeth, AstraZeneca and Pfizer, and is a member of the advisory board for AstraZeneca A/S, Denmark. LK has been a consultant for Bristol-Myers Squibb, Eli Lilly, Lundbeck, AstraZeneca, Pfizer, Wyeth, and Servier.

## Authors' contributions

All authors were involved in the conception and design of the study protocol, drafting or revising the manuscript, and have approved the final manuscript. UK and LK are the clinical investigators. UK is the sponsor and co-ordinating investigator and is responsible for inclusion of participants and research interviews. UK, LK, JW, and CG are responsible for the data analysis. All authors will participate in interpretation of results and in the writing of subsequent papers.

## Appendices

### Appendix 1. Criteria for inclusion and exclusion in the AGENDA trial

#### Inclusion criteria

• Healthy individual of both sexes. Women should preferable be in day 1–13 of her menstrual cycle at the time of randomization.

• Offspring or sibling of an ethnic Dane, with a history of psychiatric in- or outpatient care with the diagnosis of depression and who later had the diagnosis verified in a SCAN interview at the Department of Psychiatry Rigshospitalet, Denmark 2004–2009.

• Aged 18 – 60 years.

• Born in Denmark.

• European parents and grandparents.

• Able and willing to sign informed consent.

#### Exclusion criteria

• Somatical illness or other handicap, which make participation in the trial impossible.

• Daily intake of drugs interfering with corticosteroids or escitalopram, including birth control pills or any kind of corticosteroids.

• Hypersensitivity to escitalopram, dexamethasone, or human corticotrophin-releasing hormone.

• Former medical or psychological treatment for diseases in the affective or schizophrenic spectrum.

• Abuse of alcohol or psychotropic medication.

• For women: pregnancy or breastfeeding.

### Appendix 2. Assessments of prognostic factors and outcome measures in the AGENDA trial

#### Basic information

Socio-demographics, family history of psychiatric illness, Kendler's questionnaire for lifetime events in a brief Danish version [71], Schedules for Clinical Assessment in Neuropsychiatry, version 2.1[64], The Structured Clinical Interview for DSM – IV Axis II Personality Disorders [72], birth weight, height, current weight, waist-hip ratio and Edinburgh Inventory for handedness [73]. For women a pregnancy test is performed.

#### At entry and at four weeks of intervention the following variables are assessed

##### HPA-Axis

The Combined Dexamethasone Corticotrophine-releasing Hormone Test (DEX-CRH) salivary cortisol, plasma cortisol and plasma-ACTH are measured every 15 minutes from 14:00 – 18:00 hours [9].

##### Cognition

The Danish Adult Reading Test [37], Familiar faces [38], Trail Making A and B [39], Stroop test [74], Boston naming [40], Block Designs [41], Cambridge Cognitive Examination [43], Rey Auditory Verbal Learning Test [44], Rey-Osterrieth Complex Figure [45], verbal fluency for animals and letter "s" [46], Symbol Digit Modalities Test [47], Iowa Gambling Task [48], Letter-number sequencing [49], recognition of facial emotions [54], moral judgement [51,52], Mayer-Salovey-Caruso Emotional Intelligence Test [53].

##### Personality

Eysenck Personality Questionnaire [18] and NEO Personality Inventory revised, computer version [19].

##### Rating scales of mood

Hamilton Depression Scale 17-items [55] and Hamilton Anxiety Scale, 14-items [56].

##### Depressive symptoms, quality of life, perceived stress, subjective evaluations of aggression, sleep and pain

Buss-Perry Aggression Questionnaire [58], Beck Depression Inventory, 42-items [59], Side Effect Self Rating Scale by UKU-SERS-Pat [63], WHO Quality of Life [75], Cohen's Perceived Stress Scale [61] and Klepstad Visual Analogue Scale for pain, modified [57]. Sleep is evaluated by Hamilton Depression Scale and additional questions of number of night sleep hours, subjective quality of sleep on a visual analogue scale and number of interruptions of sleep.

##### Salivary cortisol

Saliva samples by Salivettes for measurements of cortisol are obtained at the clinic and during a day in the participant's usual environment.
